# Measuring tissue displacement of the anterior vaginal wall using the novel aspiration technique in vivo

**DOI:** 10.1038/s41598-017-16083-0

**Published:** 2017-11-23

**Authors:** Barbara Röhrnbauer, Cornelia Betschart, Daniele Perucchini, Michael Bajka, Daniel Fink, Caroline Maake, Edoardo Mazza, David Amos Scheiner

**Affiliations:** 1 Institute of Mechanical Systems, ETH Zurich, Tannenstrasse 3, 8092 Zurich, Switzerland; 2 Department OB/GYN, Division of Gynecology, University Hospital Zurich, Frauenklinikstrasse 10, 8091 Zurich, Switzerland; 3 Institute of Anatomy, University of Zurich, Winterthurerstrasse 190, 8057 Zurich, Switzerland

## Abstract

Little is known about the mechanical properties of pelvic floor structures and their role in the course and treatment of pelvic organ prolapse (POP). We hypothesize that *in vivo* mechanical properties of the vaginal wall are related to the appearance of POP and pre-and post-operative states. We used a suction device for intravaginal application, the aspiration device, to evaluate two *in vivo* mechanical parameters of the anterior vaginal wall, the load dependent tissue displacement and the initial displacement, by image analysis in pre- and post-menopausal women with (POP) and without (control) cystocele (POP: pre-menopausal: N = 6, post-menopausal: N = 19, control: pre-menopausal: N = 17, post-menopausal: N = 6). Mechanical parameters in women with and without cystocele and pre- and post-operative parameters were compared. Statistically significant differences were observed between the two mechanical parameters in pre- and post-operative states (P = 0.04, P = 0.03), but not between the parameters for women with and without cystocele (P = 0.92, P = 0.75). The mechanical behavior of pelvic floor structures is influenced by factors such as POP, age or estrogenization that are apparent at different length scales, which cannot be separated by the aspiration based biomechanical measurements. When comparing pre- and post-operative states of the same patient, a firmer tissue response was observed after intervention.

## Introduction

A woman’s lifetime risk of undergoing an operation for pelvic organ prolapse (POP) or urinary incontinence is 11.1%^1^. Still, little is known about the mechanical properties of pelvic floor structures and tissues which play a major role in the pathogenesis and course of POP^2–4^. In fact, corresponding findings seem to support different conclusions.

*Ex vivo* mechanical tests have been conducted using tissue strips of prolapsed and non-prolapsed vaginal wall excised during surgery or excised from female cadavers^2,3,5–12^. In uniaxial tension, prolapsed vaginal wall tissue is stiffer than control tissue^6,11^. And prolapsed vaginal wall tissue is stiffer in shear deformation compared to control tissue^12^.

Alternatively, *in vivo* methods using tactile and suction devices have been presented aiming at an intravaginal evaluation of the mechanical properties of the vaginal wall^13–15^. In contrast to *ex vivo* findings, *in vivo* stiffness of the vaginal wall is reported to be inversely correlated to the stage of POP^13,15^. Moreover, all devices have been able to discriminate between the mechanical properties in pre- versus post-operative states. The post-operative vaginal wall was reported to be stiffer than the pre-operative in patients treated by different surgical techniques, i.e. before and after mesh application, native prolapse repair or sacrocolpopexy^16–18^.

Our study intended to shed light onto the apparently contradictory *in vivo* and *ex vivo* findings with respect to the relationship between vaginal wall stiffness and POP. We used a novel suction device we have called “the aspiration device” to evaluate *in vivo* mechanical parameters of the anterior vaginal wall in pre- and post-menopausal patients with and without cystocele under optical guidance by means of a video camera. For women undergoing traditional surgical cystocele repair, mechanical parameters were compared pre- and post-operatively. It is hypothesized that the assessed mechanical parameters allow discrimination between the mechanical properties of the anterior vaginal wall of women (i) with and without cystocele and (ii) in pre- and postoperatively.

## Materials and Methods

### Study design

In this prospective, non-randomised, controlled, non-blind, interventional study in collaboration between the Department of Gynecology of the University Hospital Zurich (USZ), the Institute of Anatomy of the University of Zurich and the Center of Mechanics of ETH Zurich, pre- and post-menopausal women with cystocele (POP group) and without cystocele (control group) who were hospitalized at USZ for gynecological intervention were invited to participate (written informed consent required). In the POP group, POP repair was performed by means of native repair without implanting a mesh, such as anterior colporrhaphy, posterior colporrhaphy, or sacrospinal ligament fixation as appropriate. The control group consisted of patients without POP who underwent hysterectomy for benign gynecological condition. Exclusion criteria were age below 18 years, pregnant, breastfeeding, malignancies in the pelvis or local infections, or previous POP surgery.

### Clinical examination

The patients were examined the evening before surgery in semirecumbent position with an empty urinary bladder. To avoid artificial lubrication by examination or ultrasound gel, the aspiration measurement was taken first. Afterwards, the stage of prolapse was quantified in accordance with the POP-Q standard^19^. Clinical parameters, such as age, body mass index (BMI), parity and menopausal state were recorded. During post-operative follow-up of the POP group at 6 to 12 weeks, clinical examination and POP-Q assessment were performed, and in patients who had undergone anterior colporrhaphy the aspiration measurement was repeated.

### Aspiration device

The applied aspiration technique is based on the pipette aspiration principle^20^. The tissue of interest is elevated by a defined vacuum pressure and the resulting tissue displacement is evaluated thus providing a metric proportional to tissue compliance. The aspiration device has been used in several clinical applications^21–23^. No complications have been reported, and no risks and no harm were to be expected for the participants.

**Figure 1:**
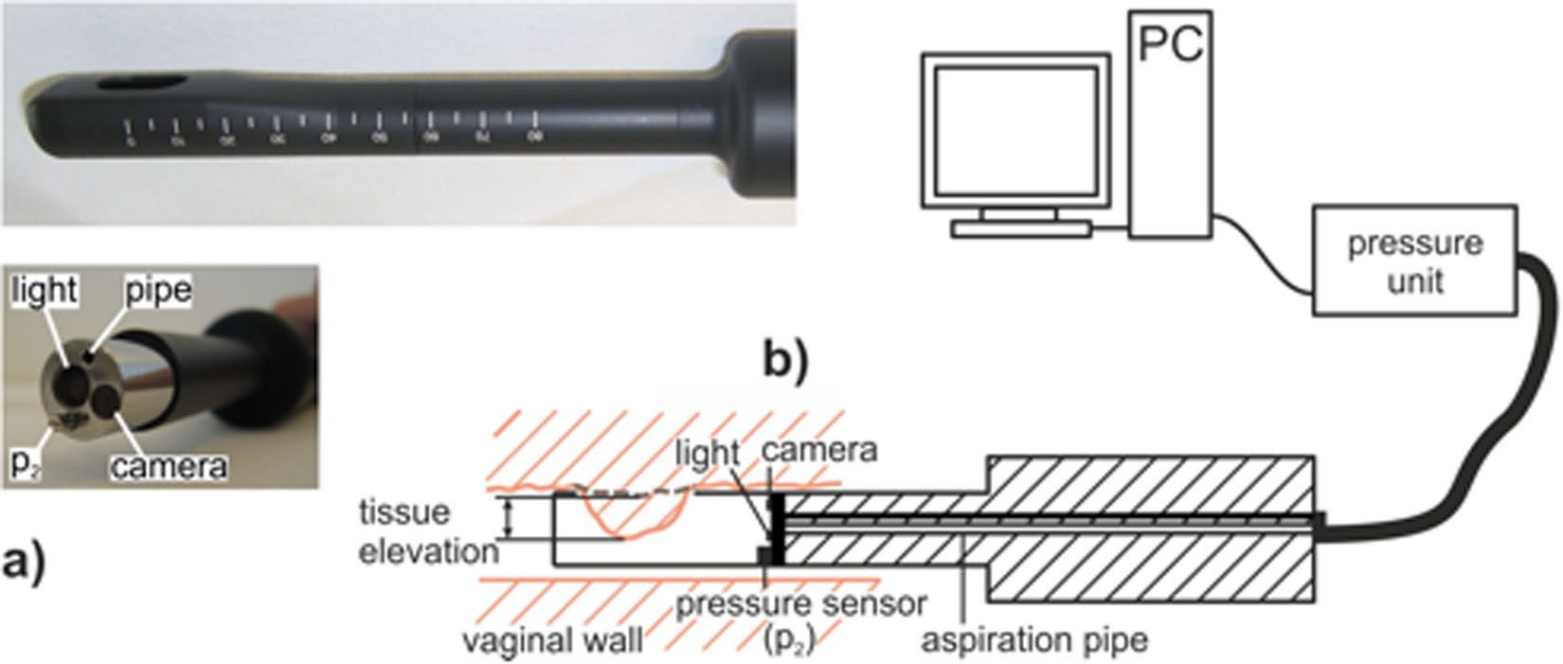
The Aspiration Device. (**a**) Aspiration probe, top: modular tip with the aspiration opening and a scale, bottom: probe with the unmounted tip, exposing the aspiration pipe, the optical fiber providing light, the camera objective and the pressure sensor (p_2_). (**b**) Transvaginal application (schematic view): The lateral opening is attached to the vaginal wall. The tip cavity is evacuated through the aspiration pipe. The current vacuum pressure is observed by the internal pressure sensor (p_2_) and controlled by the pressure unit. The camera records side view images of the intruding tissue at rest (dashed line) and at load (full line). The tissue elevation describes the difference between these two configurations.

**Figure 2:**
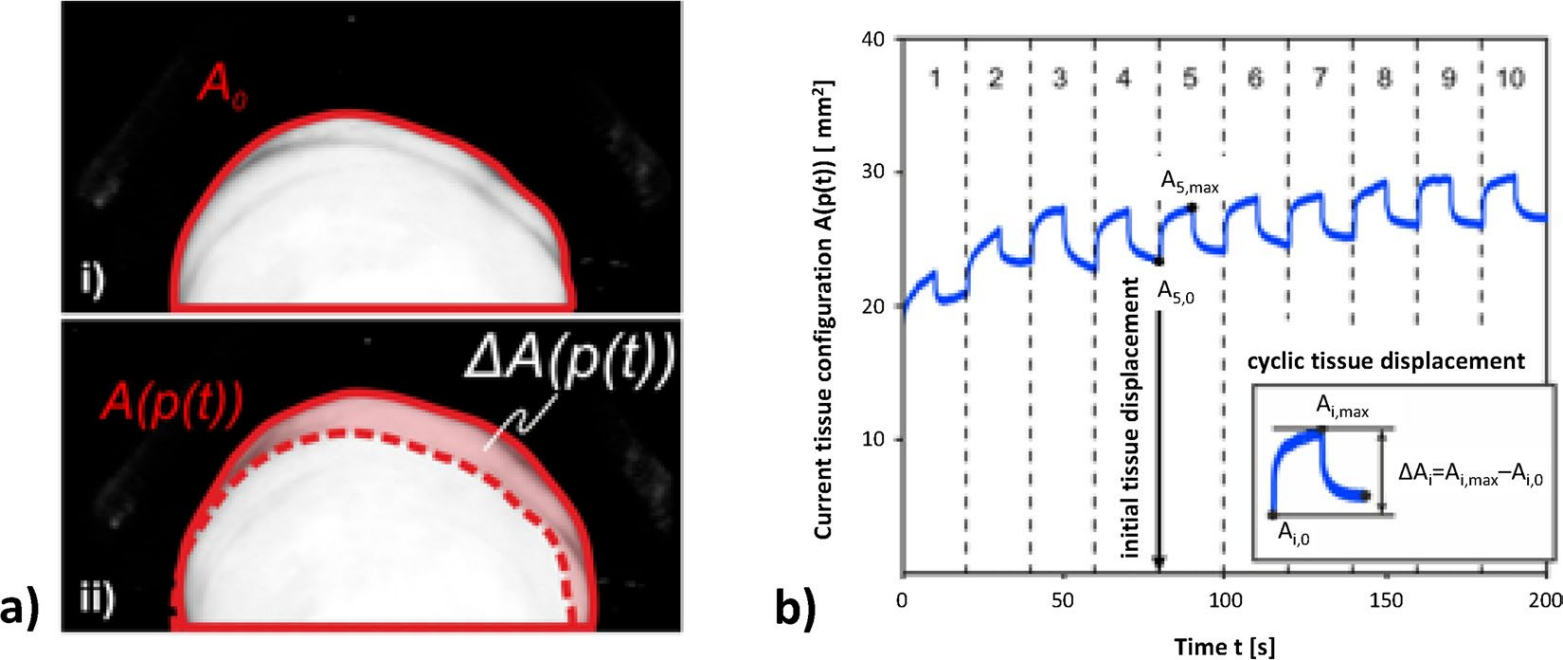
Mechanical parameters. (**a**) The area enclosed by the contour of the tissue displacement is a quantitative measure of the tissue configuration. The area enclosed by the undeformed configuration is called the reference configuration A_0_ (i). The load dependent difference of each current tissue configuration A(p(t)) with respect to the reference configuration A_0_ is called the tissue elevation ΔA(p(t)), which is indicative of tissue displacement (ii). (**b**) Evaluation of the mechanical parameters based on the history of the load dependent tissue configuration A(p(t)). Only cycles 5–10 were considered. The cyclic tissue displacement ΔA_i_ = A_i,max_ − A_i,0_ was defined as the maximum tissue elevation in each cycle. The initial displacement was defined as the initial unloaded tissue configuration in cycle 5.

We used a probe design specifically developed for vaginal application. The aspiration setup consisted of the measurement probe, a pressure unit, and a personal computer with the appropriate software (Fig. 1a and b). The aspiration probe (AP) (Fiberoptic P & P AG, Spreitenbach, Switzerland) was a slender tube with a modular tip, serving as a vacuum cavity (Fig. 1a). A camera and a pressure sensor (p_2_) were mounted within the cavity. At its distal end, the tip had a lateral aperture shaped as a long-hole (13.5 mm × 8.5 mm). For *in vivo* measurements, the probe was applied intravaginally such that the aperture was placed on the anterior vaginal wall (Fig. 1b). The vacuum within the aspiration cavity, i.e. the load applied to the tissue, was provided by the pressure unit. In addition to electronic components, the pressure unit also included a vacuum container, a diaphragm gas pump (Type: NMP 830 KNDC, KNF Neuberger AG, Balterswil, Switzerland), two valves (Type: VDW21-5G-3-01F-Q, SMC Pneumatik AG, Weisslingen, Switzerland), an internal pressure sensor (Type: XFGMC-3025KPGSRH, Pewatron AG, Zurich, Switzerland), and a tubing system. The vacuum container had a capacity of 0.01 m^3^ evacuated by the pump to a controlled constant vacuum pressure. Controlled by the valves, the aspiration cavity was either connected to the vacuum container or to the environment, subjecting the tissue to the vacuum pressure or releasing it, respectively. The actuators, the pump and the two valves were computer-controlled, realizing a defined load history of the air pressure (that is suction) p(t) [N/mm^2^] acting on the tissue. The respective software was programmed with LabVIEW (National Instruments, Austin, Texas, USA). The integrated camera recorded a side view image sequence of the current tissue configuration at a frame rate of 20 images/s and allowed a continuous visual control (Fig. 2a). The measurement output consisted of a continuous pressure signal and a corresponding image sequence, which needed further processing, in order to quantify the tissue displacement. The camera images were further used to quantify the visual appearance of the vaginal wall through the nature of rugae. For this purpose, we defined index taking values of 1–3, 1 indicating that no rugae were visible on the anterior vaginal wall, 2 corresponding to intermediate, and 3 to distinct rugae.

### Aspiration protocol

Aspiration measurements were performed on the anterior vaginal wall, 5 cm proximal to the hymen in the region close to the bladder neck. This location was chosen as the bladder neck is directly affected by an anterior vaginal wall prolapse and as the vaginal wall is less wrinkled (rugae) leading to more reproducible measurements. All measurements were done by the same experienced gynecologist. The vacuum container was evacuated to −25 mbar (relative to the atmospheric pressure), a pressure level which was, on the one hand harmless for the patient, and on the other hand high enough to obtain a visible tissue deformation. The tissue was loaded (−25 mbar) and released (0 mbar) cyclically by holding each level for 10 s. One measurement was performed per patient and consisted of ten such cycles, lasting for a total of 200 s. With respect to measurement failure, we differentiated between technical failure such as malfunction of technical components and procedural failure such as insufficient vacuum or the the vaginal wall touching the inner surface of the aspiration cavity.

### Data processing

Programs for data analysis were written with Matlab (The Mathworks Inc., Natick, Massachusetts, USA). The image sequence recorded by the camera documented the current, deformed tissue configuration dependent on the defined pressure history p(t). The tissue displacement was determined from the load dependent change of the tissue configuration. The current tissue configuration at time-point t [s] subject to the current pressure p(t) was quantified by the two-dimensional projected area A(p(t)) [mm^2^] enclosed by the contour of the tissue displacement (Fig. 2a).

### Mechanical parameters

The mechanical response measured with the aspiration experiment is influenced by the mechanical properties of tissue below the surface, down to 1–2 times the radius of the aspiration device opening^24^. In the present case, this reaches the full vaginal wall thickness including (to some extent) the influence of fascial tissue. In accordance with the literature, an intact integration of the vaginal wall within the pelvic floor and an intact microstructure were assumed to be associated with a compact, dense unloaded configuration and a stiff mechanical response^13,15^. Moreover, scar tissue formation and a change in anatomical boundary conditions, which are present after a surgical intervention were expected to increase the structural stiffness of the vaginal wall. A stiffness-like mechanical parameter was evaluated based on the history of the current tissue configuration A(p(t)). The difference between any current tissue configuration A(p(t)) and a defined reference configuration A0 was called tissue elevation ΔA(p(t)) = A(p(t)) − A_0_ (Fig. 2a). The cyclic tissue displacement was defined as the maximum tissue elevation per cycle ΔA_i_ = A_i,max_ − A_i,0_ as shown in Fig. 2b, where the indices i, max and 0 denote the cycle number, the cyclic maximum and the cyclic initial tissue intrusion. It was observed, that the cyclic tissue displacement during the initial cycles was weakly reproducible, comparable to preconditioning effects, known for biological tissues^25^. Thus, cycles 1–4 were not considered for parameter evaluation. The median cyclic tissue displacement of cycles 5–10 was called *tissue displacement ΔA*_*5–10*_, a parameter representing the local structural and material stiffness of the vaginal wall. A representative unloaded state of the vaginal wall was defined by the initial tissue configuration in cycle 5, called *initial* displacement *A*_*5,0*_ (Fig. 2b). Tissue displacement and initial displacement were analyzed for differences between POP and control, pre- and post-menopausal state, and pre- and post-operative measurements.

### Grouping of participants

The participants were subdivided into a pre- and post-menopausal group. Postmenopause was defined as absence of menstruation for a minimum of 12 months. Perimenopausal women were considered as premenopausal. Cystoceles stage 0 and I (POP-Q) were considered as control, and stage >I as POP. Systemic or local hormone treatment was also recorded but not considered for the present study as this would have led to even smaller group sizes.

### Statistics

Statistical evaluation was carried out using Matlab. The data was treated anonymously. Descriptive statistical data was presented as mean, standard deviation (SD), and range (minimum – maximum). The mechanical parameters in the POP and control group were compared using the Mann-Whitney U test. Changes due to a surgical intervention were analysed using the Wilcoxon signed rank test. P values < 0.05 indicated statistical significance (two-sided). Due to the lack of preliminary data for the aspiration technique of the anterior vaginal wall with our device, no sample size calculation was feasible.

This trial was performed in accordance with the approved protocol obtained from the local Cantonal ethics committee of Zurich (StV 11/2009). This study is registered with ClinicalTrials.gov, number NCT01042470 (date of registration: January 4, 2010).

## Results

### Basic characteristics

Between November 2010 and February 2012, 48 women were recruited (Table 1), with all having a pre-operative assessment with the aspiration technique. Mean age was 58.2 years (SD 13.4, range 38.2–83.7), mean BMI 25.9 (SD 4.0, range 18.1–35.3), and mean parity 1.6 (SD 1.3, range 0–5). In 14 women, both pre- and post-operative measurements were performed. In the pre-menopausal subgroup, patients with POP had statistically significantly a higher parity than patients in the control group (1.3 ± 1.4 vs. 3.0 ± 1.3, P = 0.03). In the post-menopausal subgroup, patients with POP were with 71.2 ± 8.4 years statistically significantly older than patients in the control group (61.0 ± 8.8, P = 0.03).

**Table 1:** Basic characteristics (N = 48).

	Pre-menopausal			Post-menopausal		
	Control	POP	P value^a^	Control	POP	P value^a^
N	17	6		6	19	
Age (years)	46.6 ± 4.6 (38.2–55.8)	47.0 ± 5.3 (40.3–56.0)	0.97	61.0 ± 8.8 (52.8–72.9)	71.2 ± 8.4 (38.2–72.9)	0.03
BMI (kg/m^2^)	25.6 ± 3.7 (20.0–32.6)	28.4 ± 2.7 (24.6–32.5)	0.09	24.7 ± 5.0 (18.1–30.5)	25.7 ± 4.1 (18.4–35.3)	0.70
Parity	1.3 ± 1.4 (0–4)	3.0 ± 1.3 (2–5)	0.03	1.2 ± 1.3 (0–3)	1.6 ± 1.1 (0–4)	0.49
Rugae^b^	2.1 ± 0.6 (1–3)	2.3 ± 0.8 (1–3)	0.42	1.4 ± 0.5 (1–2)	1.6 ± 0.8 (1–3)	0.78
POP-Q Aa (cm)	−2.6 ± 0.5 (−3.0-(−2.0))	2.0 ± 1.3 (0.0–3.0)	P < 0.0001	−2.5 ± 0.5 (−3.0-(−2.0))	2.2 ± 1.0 (0.0–3.0)	P < 0.0001
POP-Q Ba (cm)	−2.6 ± 0.5 (−3.0-(−2.0))	2.0 ± 1.3 (0.0–3.0)		−2.5 ± 0.5 (−3.0-(−2.0))	2.7 ± 1.8 (0.0–8.0)	
POP-Q Stages (separated for each group)			P < 0.0001			P < 0.0001
0	10	0		3	0	
1	7	0		3	0	
2	0	2		0	5	
3	0	1		0	4	
4	0	3		0	10	

Data are expressed as mean ± standard deviation and range (minimum – maximum), or number of patients. ^a^Mann-Whitney U test between control and POP. ^b^Rugae were visually assessed (1 no rugae, 2 intermediate and 3 distinct rugae).

### Aspiration measurement: Pre- versus post-operative

Statistically significant differences were observed between mechanical parameters for pre- and post-operative measurements: both tissue displacement and initial displacement were smaller after POP surgery (ΔA_5–10_ P = 0.04, A_5,0_ P = 0.03) as presented in Table 2.

**Table 2:** Aspiration measurements. Pre- and post-operative mechanical properties (N = 14).

	pre-operative	post-operative	P value^a^
Tissue displacement^b^ [mm^2^]	4.6 ± 1.9 (1.5–8.1)	3.5 ± 0.8 (1.7–4.8)	0.04
Initial displacement^c^ [mm^2^]	30.4 ± 14.3 (15.9–63.8)	20.5 ± 12.7 (7.7–52.9)	0.03

Data are expressed as mean ± standard deviation and range (minimum—maximum), or number of patients. ^a^Wilcoxon signed rank test. ^b^Tissue displacement ΔA_5–10_, defined as the median value of the maximum tissue elevation in cycles 5–10. ^c^Initial displacement A_5,0_, defined as the initial tissue configuration in cycle 5.

### Aspiration measurement: POP versus control

No statistically significant differences between the mechanical parameters tissue displacement (ΔA_5–10_) and initial displacement (A_5,0_) could be assessed for women with (POP) and without (control) cystocele, neither for pre-menopausal (ΔA_5–10_, P = 0.92; A_5,0_, P = 0.75) nor for post-menopausal (ΔA_5–10_, P = 0.63; A_5,0_, P = 0.15) women (Table 3).

**Table 3:** Aspiration measurements. Pre-operative mechanical properties in participants without (control) and with (POP) cystocele (N = 48).

	Pre-menopausal			Post-menopausal		
	Control	POP	P value^a^	Control	POP	P value^a^
N	17	6		6	19	
Tissue displacement^b^ [mm^2^]	4.5 ± 1.7 (1.7–7.6)	4.3 ± 1.8 (2.5–7.7)	0.92	4.2 ± 1.5 (1.8–6.2)	4.8 ± 2.2 (1.5–8.9)	0.63
Initial displacement^c^ [mm^2^]	31.7 ± 15.2 (15.2–66.4)	27.6 ± 10.7 (16.3–43.5)	0.75	22.1 ± 10.4 (10.2–38.4)	32.7 ± 15.0 (15.9–63.8)	0.15

Data are expressed as mean ± standard deviation and range (minimum—maximum), or number of patients. ^a^Mann-Whitney U test. ^b^Tissue displacement ΔA_5–10_, defined as the median value of the maximum tissue elevation in cycles 5–10. ^c^Initial displacement A_5,0_, defined as the initial tissue configuration in cycle 5.

No measurements had to be excluded due to procedural failure (procedural failure rate 0%). Technical failure was due to computer-related problems saving the image sequence. The technical failure rate was <10%.

## Discussion

The aim of this study was to detect *in vivo* differences in the biomechanical properties of the anterior vaginal wall in patients with and without cystocele and before and after anterior colporrhaphy. Using a novel and innovative technique, the aspiration device, our present aspiration protocol allows detection of such differences between the pre-operative and post-operative situation, but not between women with and without cystocele.

Our results are in accordance with the findings of Werbrouck *et al*. who reported no significant differences in the mechanical properties of the vaginal wall in women with and without POP^14,16^. Conversely, Epstein *et al*. reported correlations between the mechanical properties of the vaginal wall and the stage of prolapse^15^. In contrast to our study, Epstein *et al*. and Werbrouck *et al*. applied high levels of suction pressure up to −300 mbar (compared to −25 mbar in our study) resulting in a high level of applied forces of up to 1.57 N (compared to 0.25 N in our study). Due to the nonlinear stress strain relationship which is characteristic for most soft biological tissues, a reason for their need of such supraphysiological forces could be the implied prestrain on the vaginal wall caused by a high contact force and the use of a speculum. In our study, a vacuum pressure of −25 mbar was seen to represent an upper bound to reliably avoid the tissue touching the inner wall of the cavity of the aspiration probe. With respect to the tissue reference configuration and according boundary conditions, we aimed at an unloaded configuration closely reproducing the physiological *in vivo* conditions. The inhomogeneous, wrinkled structure of the vaginal wall with the typical rugae and the lubrication represented major sources of uncertainty influencing the reproducibility of the measured data. In contrast, Epstein *et al*. defined a standard protocol to set up an altered, but more reproducible reference configuration^15^. They inserted a speculum, pre-stretching the vagina, and flattening the rugae. The measurement position was chosen as 2 cm proximal to the hymen, and thus more distal as compared to our protocol and closer to the speculum. The measurement site was cleaned and dried with a swab, removing the lubricating film and thus normalizing the friction behavior. Hence, in particular, mesostructural influences such as rugae and lubrication were reduced. The application of uncontrolled, unphysiological boundary conditions might induce systematic bias in the determination of the biomechanical properties. This approach was therefore avoided in our protocol. Recently, Parkinson *et al*. demonstrated another approach to measure the vaginal pressure profile, using an optical-fiber-based instrumented speculum for distributed real-time pressure measurement of the vaginal pressure profile in sheep^26^. They found higher pressure profiles for nulliparous than for parous sheep and observed an increased tissue laxity in the upper anterior vagina for parous sheep.

How do we interpret the findings from a biomechanical point of view? The structure of the vagina can be sub-divided into different length scales. At the *macroscale*, the vagina is embedded within the pelvic floor, determining its global boundary conditions (anatomy). At the *mesoscale*, the surface of the vaginal wall has a wrinkled and layered structure consisting of rugae. The *microscale* is mainly characterized by the fibrous constituents of the extracellular matrix, i.e. collagen and elastin. Among other factors, POP, estrogenization, and age influence the vaginal wall at all these length scales. POP is a macroscale change of the pelvic floor anatomy, related to vaginal wall laxity and decreased vaginal wall integrity^19,27^. Moreover, POP is associated with alterations of the microstructure, i.e. of the collagen metabolism and the content of normal elastin^27–29^. The meso- and microstructure are influenced by estrogen and age. They determine the appearance of the rugae, the collagen maturation and the content of elastin^27,30^. The relative contribution of each factor to the actually measured mechanical parameter depends on the sensitivity of the aspiration measurement to phenomena at the different length scales. The current procedure is not sensitive to changes at one single length scale. However, significant changes influencing all length scales, such as surgical cystocele repair, can be addressed with the present protocol.

In contrast to *in vivo* measurements, *ex vivo* tests exclude anatomic influences (macrostructure), such as the level of vaginal wall integrity. Testing after cadaver dissections can provide excellent detail on structural morphology, but biomechanical properties can be distorted by post-mortem or embalming processes. *In vivo*, the prolapsed, less integrated and slack vaginal wall leads to a decreased structural stiffness compared to the intact, well integrated vaginal wall^13,15^. *Ex vivo*, all slackness is removed, leading to a stiffened material response^6,11,12^. These differences in the tissue reference configuration are probably responsible for the divergent findings in the current literature.

When comparing *in vivo* and *ex vivo* techniques, major limitations of *ex vivo* studies are the restricted availability of non-prolapsed human vaginal wall tissue and the minor relevance particularly of uniaxial tensile tests, due to non-physiological loading and boundary conditions^5,31^. In contrast, non-invasive *in vivo* measurement techniques represent promising tools for POP diagnosis and prevention^15,18^. Based on the mechanical properties of pelvic tissues, the risk for a recurrent prolapse will be reduced through case specific treatment (native tissue or prosthetic mesh repair) or a more conscious selection of prosthetic materials^3,5,31^. Moreover, follow-up observations of mechanical parameters should help to evaluate the long-term surgical outcome^17,18^.

The strength of our study with the aspiration device is that its vaginal application caused no discomfort, no pain, and no complications. Compared to procedural failure rates of approximately 20% and >50% in other studies^14,15^, our aspiration protocol demonstrated an outstanding reliability (procedural failure rate of 0%). A significant and very important value was seen in the use of a camera, which is a unique characteristic of the present suction device. The camera provided visual feedback which enabled us to guarantee safety and to exclude procedural measurement failure. Furthermore, visual analysis of the tissue configuration allowed the challenges related to the influence of rugae and the selection of a reference configuration to be identified.

The unbalanced groups are one limitation of this study. On the one hand, pre-menopausal women with cystocele were quite rare and on the other hand, post-menopausal controls were less frequently hospitalized in the USZ, leading to a selection bias.

We believe that a characterization of the pelvic floor mechanics related to POP needs to address processes at different length scales and at the same time needs to be able to differentiate between them, - a challenging requirement that has not yet been reached by any of the currently applied techniques. In this context, the present study might contribute to a further improvement of currently available, together with the development of alternative, *in vivo* techniques which are still the subject of fundamental research. In particular, the combination of aspiration experiments with high resolution tissue imaging might provide direct insight on the level of deformation of each tissue structure contributing to the mechanical properties of vaginal wall.

## Conclusions

Our novel aspiration device gives a visually monitored insight into the biomechanical behavior of the anterior vaginal wall. So far, the aspiration technique has enabled the detection of differences in the mechanical properties of the vagina in patients before and after cystocele repair. For future research, there is a need to gain more insight into the relationship between biomechanical properties of pelvic floor structures and POP. Moreover, it would be of interest to classify findings according to demographic parameters like specific age groups, parity status, ethnic group, hormonal status and/or other varying pelvic floor conditions. Further research of the biomechanical properties of the vaginal wall and the pelvic floor are important, in particular with regard to recurrences after native repair for POP surgery and the actual debate on alloplastic meshes for POP repair.
